# EnduroBone: A 3D printed bioreactor for extended bone tissue culture

**DOI:** 10.1016/j.ohx.2024.e00535

**Published:** 2024-04-18

**Authors:** Paula Gustin, Anamika Prasad

**Affiliations:** a Department of Biomedical Engineering, Florida International University, Miami, FL, United States; b Department of Mechanical and Materials Engineering, Florida International University, Miami, FL, United States; c Biologcial Science Institute, Florida International University, Miami, FL, United States

**Keywords:** 3D bioreactor, Bone, Long-term culture, Cell viability

## Abstract

Studies of the effects of external stimuli on bone tissue, disease transmission mechanisms, and potential medication discoveries benefit from long-term tissue viability ex vivo. By simulating the in-vivo environment, bioreactors are essential for studying bone cellular activity throughout biological processes. We present the development of an automated 3D-printed bioreactor EnduroBone designed to sustain the ex-vivo viability of 10 mm diameter cancellous bone cores for an extended period. The device is supplied with two critical parameters for maintaining bone tissue viability: closed-loop continuous flow perfusion of 1 mL/min for nutrient diffusion and waste removal and direct mechanical stimulation with cyclic compression at 13.2 RPM (revolutions per minute) to promote cell viability which can lead to improved tissue stability during ex vivo culturing. The bioreactor addresses several limitations of existing systems and provides a versatile open-source platform for bone cancer research, orthopedic device testing, and other related applications. To validate the bioreactor, fresh swine samples were cultured ex-vivo, and their cell viability was determined to be maintained for up to 28 days. Periodic cell viability assessment through live/dead cell staining and confocal imaging at the start (0 days) and at several time points throughout the culture period (7, 14, 21, and 28 days) was used to demonstrate *EnduroBone* effectiveness in sustaining bone cell health for the extended period tested.

## Highlights

• Design, 3D printing, and assembly for an affordable bioreactor for ex-vivo bone tissue culture.
• Incorporates both flow and mechanical loading to mimic physiological loading .
• Demonstrates applicability of the system for long-term tissue viability up to 28 days.
• Addresses the limitations of commercial bioreactors ivia versatilite open design, y.
• Utilizes3D printing methods and easily accesible components for replication.
• Provides an open-source platform to advance bone tissue engineering research through long-term culture.

## Graphical abstract

**Figure:**
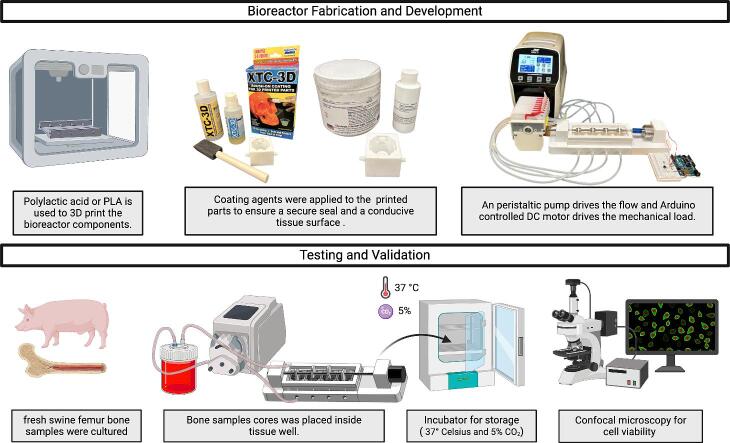

## 1 Specifications table

**Table:** 

Hardware name	3D-Printed Bioreactor for Ex-Vivo Tissue Culture
Subject area	Bone Tissue Engineering, Biomaterials
Hardware type	Biological sample handling and preparation
Closest commercial analog	MechanoCulture TR
Open-source license	This work is licensed under a Creative Commons Attribution 4.0 International License.
Cost of hardware	$ 3,138.35 USD
Source file repository	https://dx.https://doi.org/10.17632/w496dkygx8.1

## 2 Hardware in context

Studying the development of bone biology in healthy and diseased states is crucial in biomedical research for wide-ranging applications, including probing disease progression, studying drug efficacy, and developing new biomaterials and drugs. An ex-vivo 3D bone tissue culture maintained in an appropriate bioreactor provides an excellent platform for such research. They also serve as ethical pivots away from traditional animal testing. Furthermore, with the new FDA Modernization Act 2.0 [1], there is now a greater push toward viable and effective alternatives to animal testing. The primary function of such systems is to provide a controlled physiological environment for tissue survival through precise regulation of essential parameters such as temperature, pH, nutrient flux, and metabolic waste management. Additional to these parameters is the need to mimic the dynamic strain environment of bone tissue to promote cell differentiation and mineralization and maintain larger 3D tissue over a longer period. Several earlier studies have shown the critical influence of loading on bone tissue scaffolds [2], [3], [4], [5], [6], [7], [8], [9]. Furthermore, extended culture periods are preferable but can be challenging to maintain for bone tissue due to their dense and compact structure [10].

Despite the critical role of such combined stimulation to bone biology, an integrated bioreactor with perfusion and mechanical stimulation for 3D bone cultures is either lacking or significantly expensive, limiting its wide use. MechanoCulture TX and TR (Cell Scale, Canada) are such systems that can provide perfusion shear together with programmable hydrostatic compression up to 500 kPa to samples placed in tissue wells for different applications [11], [12], [13], [14]. The transparent culture wells enable real-time imaging and are autoclavable for storage in an incubator. These systems, however, are expensive for wide application (MechanoCulture TX costs > 20,000 USD). Furthermore, the load is indirectly applied to the bone tissue sample through pneumatic compressing of the liquid medium filling the culture well. Other commercial bone tissue bioreactors offer only basic physiological control using a continuous perfusion system without compressive mechanical stimulation. For example, OsteoGen bioreactor systems (Tissue Growth Technologies) can simultaneously apply shear pressures via flow perfusion up to 12 samples. Multiple such perfusion-based systems, commercial or otherwise, have been used for 3D tissue culture to understand the role of growth factors, hormones, inflammatory reactions, antimicrobial therapy, bone ingrowth, and cancer metastasis [15], [16], [17], [18], [19], [20], [21].

Most of the previous studies have challenges, such as being expensive in the case of commercial reactors, lacking optimum mechanical environment for bone tissue such as in perfusion-only setups, lacking capability for long-term storage of larger bone tissue samples, or lacking design files for replication of the bioreactor by others. The advent of cost-effective 3D printers has further opened up opportunities for accessible and sharable science, even with complex designs, and includes bioreactor design.

Moving forward, there is thus a need for a compact, affordable, and open-source bioreactor device that combines programmable perfusion flow and tunable mechanical loading that can be placed inside a culture chamber for long-term tissue maintenance and follow-up testing. Such a design can address the current research needs, and its open-source design will promote future upgrades for the community. The present work addresses these challenges and introduces an innovative, cost-effective, 3D printable bone tissue bioreactor model named *EnduroBone* that breaks these constraints, setting a new standard for bone tissue research and regenerative applications. The key features of *EnduroBone* perfusion flow to deliver nutrients, remove waste, and shear to bone tissue, strain-controlled compressive mechanical loading to simulate long-term cell viability up to 28 days for long-term 3D bone tissue storage. Another feature is the choice of material used, which makes the design 3D printable, sterilizable, and storable in the humid environment of the culture chamber. The benefit in comparison with the commercial MechanoCulture system is that the *Endurobone* bioreactor will apply mechanical loading directly onto the bone tissue. The biggest advantage of the new design compared to the perfusion systems used in several earlier studies [15], [16], [17], [18], [19], [20], [21] will be the presence of dynamic mechanical loading, which is critically needed for the extended culturing of bone tissue. Hence with the design of *EnduroBone* bioreactor publicly available through the current work, a wider research community can readily and affordably replicate the bioreactor and thus will make bone-related research more inclusive, affordable, and user-friendly.

## 3 Hardware description

**Fig. 1:**
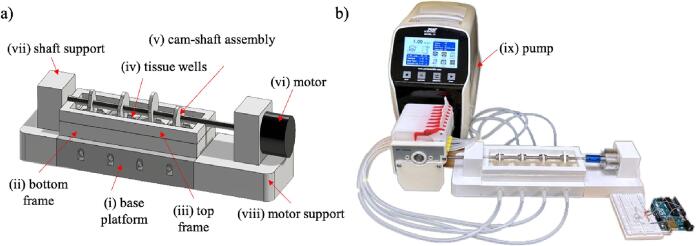
EnduroBone Bioreactor: (a) Schematic representation of the bioreactor device illustrating the cylindrical motor connected to the axle housing four evenly spaced cams over the tissue wells. (b) Actual setup demonstrating the implementation of the described schematic components.

Fig. 1 shows the *EnduroBone* bioreactor device schematic (Fig. 1a) and as-build setup (Fig. 1b). The device components include (i) a base platform, (ii) a bottom frame, (iii) a top frame, (iv) tissue wells, (v) a cam-follower assembly, (vi) a motor attached to the axle of the cam-follower assembly, (vii) shaft supports, (viii) motor support, and (ix) an external pump. The base platform houses multiple device components, including the top and bottom frame and tissue well. Each tissue well is designed to hold a cylindrical bone sample 10 mm x 10 mm long and has two side openings for inflow and outflow of nutrient media. The top frame also has openings aligned with each of the four wells. The cam-follower assembly is designed to simultaneously apply cyclical compressive load to the bone tissue housed in each tissue well, with the motor driving the assembly. The motor used for driving the cam-follower assembly is a DC 12 V 20 RPM gear motor. The peristaltic pump flow drives the nutrient media through the tissue wells. The bottom framework has a crevice that securely attaches a transparent plastic layer to preserve the nutrient media, which is held in place by the top frame. In its current form, the above design is the outcome of several design iterations through previous and ongoing research of the team [22], [23].

**Fig. 2:**
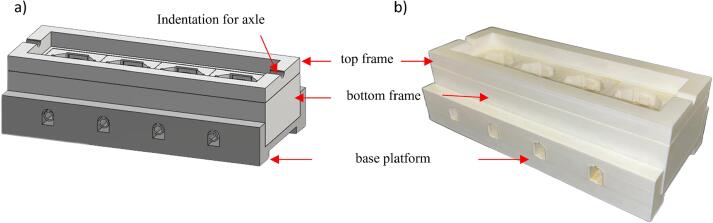
(a) Schematic of and (b) as-built setup demonstrating the integration of components for the base platform and its frames. The base platform features rail gaps for securing the top frame and the top frame has circular indentation to accommodate the axle of the cam-follower.

**Fig. 3:**
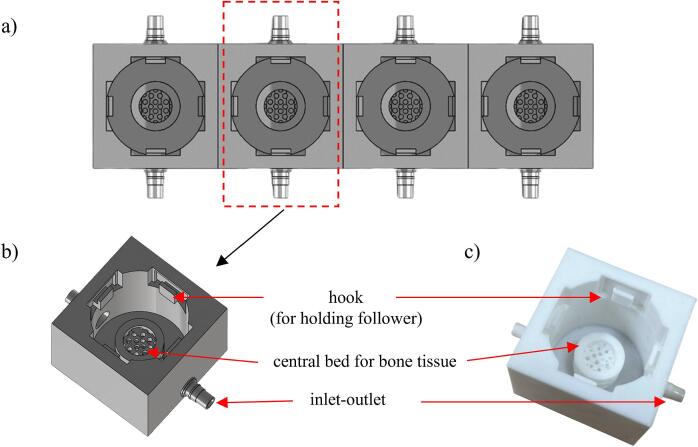
(a) The linear arrangement of identical tissue wells on the base platform within the top frame, each equipped with inflow-outflow channels for nutrient media and a porous base for continuous nourishment of bone samples. (b) Schematic depiction of tissue well with raised edges, hooks for secure bone sample placement, and integrated hollow extrusions for nutrient media flow. (c) As-built setup showing the printed design of the tissue well, incorporating the described features.

Fig. 2 shows a close-up view of the base platform and its top and bottom frame schematic and as-built. Rail gaps along the edges of the base platform are used to hold the top plate securely. Circular indentations are included in the top frame to hold the axle of the cam-follower. Fig. 3a shows the schematic plan view showing four tissue wells linearly placed. The detailed schematics and as-built for one of the wells are shown in Fig. 3b and 3c, respectively. The wells were designed with raised central bed to hold the bone sample in place during loading. Each of the four edges of the well has hooks to hold the follower of the cam-follower assembly (discussed later). The inlet–outlet opening on two opposite ends creates a flow path for nutrient media for tissue nourishment and shear stress application. Plastic hoses connect these inlet–outlet openings to an external peristaltic pump for driving the flow (Fig. 1b).

**Fig. 4:**
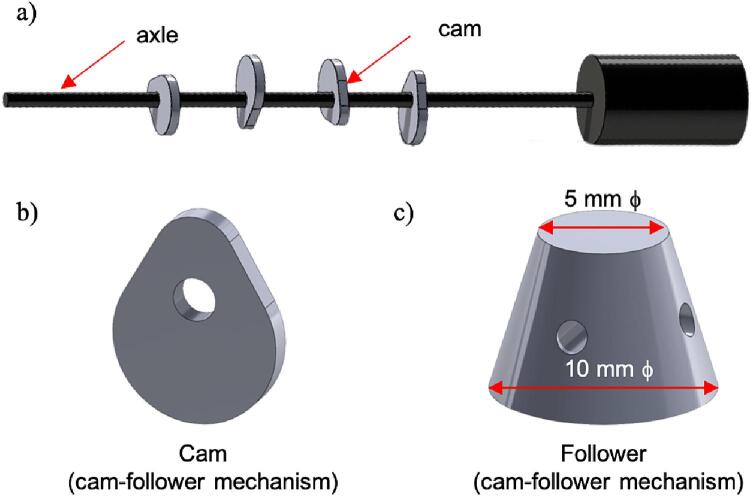
Cam-follower mechanism was used for precise force application to bone samples showing the (a) cams attached on the axle for rotation using a DC motor, (b) teardrop-shaped cam geometry, and (c) follower geometry.

Fig. 4 shows the components of the cam-follower assembly. The cam-follower ’s evenly spaced cams arranged on its axle are aligned to position directly above the bone samples sitting in tissue wells for simultaneous application of force (Fig. 4b). The unique rounded teardrop shape of the cam with an offset hole for the motor axle (Fig. 4b) was designed to apply cyclic compression loading [23]. The cam is in contact with a follower of the cam-follower assembly, which moves down to apply the load to the bone (Fig. 4c). The follower has a conical shape with the 5 mm end in contact with the cam, gradually increasing to 10 mm diameter to match the diameter of the bone sample for uniform load application. The holes in the four directions of the follower of the cam-follower assembly allow rubber bands to pass through them and attach to the hook of the tissue well to secure it in place above the bone. Using a high-torque, low-rpm (20 rpm) 12 V DC motor, the cam’s rotation engages its flat follower, exerting periodic pressure on the cultured sample. During a single rotation cycle, the follower of the cam-follower assembly undergoes oscillations, creating cyclic mechanical loading on the bones. Adjustments to the motor rpm and cam geometry (radius and offset) parameters facilitate the modulation of peak force and loading frequency. Additional details for the mechanical load calibration are provided in Section 2.1.

The base frame, multi-chamber tissue well, and the cam and follower components of the loading assembly were 3D-printed. CAD models were created in STL file format and converted into gcode using CURA (Ultimaker CURA 5.3) software for printing on an Ender 5 plus 3D printer (Creality 3D Technology Co, China). Additional details of material and print instructions will be detailed later under build instructions.

### 3.1 Pump selection

Achieving perfusion within a compact experimental setup is crucial for accurate fluid transfer and shear actuation. This pump is programmable and capable of providing flow rates ranging from 0.005 to 46 mL/min per channel. The MC-8 8-channel pump head ensured accurate fluid transfer and dispensing, while the pump’s 10 stainless steel rollers and motor speed range of 0.1 to 150 rpm contributed to its efficient operation. The pump housing is made of durable ABS engineering plastics, making it compatible with various fluids. Our experimental setup using this pump proved to be a dependable and effective way to achieve flow perfusion. Alternative lower-cost pumps can also serve the purpose used in earlier iterations of the bioreactor [22], [23].

### 3.2 Material selection

The *EnduroBone* bioreactor device was printed using polylactic acid (PLA) (HATCHBOX), a popular 3D printing material. All components, including the base platform, multi-component frame, tissue wells, motor holder, and cam-follower assembly, were carefully crafted to ensure the structural integrity of the print. The choice of PLA was guided by the fact that it is non-toxic, proven to be biocompatible, and promotes cell growth [24], [25], making it acceptable for accommodating bone samples within the bioreactor. Furthermore, since a sealant was used over PLA, as described later, the PLA is not expected to interact directly with the nutrient media. Polysulfone (PSU) filament is an alternative material choice, known for its biocompatibility, exceptional mechanical properties, and capability to withstand high temperatures, making it an excellent choice for sterilization within an autoclave [21]. However, since PSU filament requires a printing temperature of 350 °C, it requires access to a 3D printer capable of handling high printing temperatures, which was not feasible with the equipment at our disposal. Another common alternative that can be considered is acrylonitrile butadiene styrene (ABS) due to its higher temperature resistance than PLA and its ability to be printed using affordable 3D printers. However, ABS is not recommended for direct tissue applications except ABS-M30i [26] and is not compatible with steam sterilization [27], thus making it an unsuitable choice for application without access to alternatives such as gamma radiation.

A sealant XTC-3D (Smooth-On Inc., USA) was used on printed parts to secure the nutrient media perfusion seal. The sealant is expected to successfully maintain a favorable environment for bone tissue during perfusion and storage by preventing leakage [25]. Additionally, the inside of each tissue well was coated with polydimethylsiloxane (PDMS) Sylgard-184 (Electron Microscopy Sciences). Finally, the tissue wells were rinsed six times in phosphate-buffered saline (PBS) (Thermo Fisher Scientific) before being used as an additional cleaning measure to eliminate any remaining cytotoxic monomers, following earlier protocols [25]. The combined properties of PLA’s biocompatibility and the sealing efficacy of XTC-3D sealant and PDMS Slygard-18 make this bioreactor a reliable tool for carrying out cell culture studies and advancing bone tissue research.

### 3.3 Strain environment

The frequency and duration of the loading profile needs can vary depending on the tissue and specific application. Short-duration dynamic loading is beneficial for bone adaptation and cellular viability [28] Strain levels between 2000 and 4000 μɛ support bone adaptation in various animal species, while strains exceeding 7000 μɛ can cause bone failure [29]. Low-magnitude high-frequency (LMHF) loading is effective in promoting bone growth and preventing bone loss in space environments and with aging [30]. Hence, it can be essential to calibrate the strain environment of the loading setup. The target strain stimulus for the cam-follower mechanism was between 2000 and 10,000 μɛ.

## 4 Design files summary

The detailed instructions for building the design are provided in this section, with Table 1 providing the CAD files and Table 2 listing all materials needed for the following build instructions. The crucial components of the *EnduroBone* bioreactor are 3D printed, while the other necessary hardware and electronics can be easily obtained from standard suppliers.

**Table 1:** The table detailing the files to assemble the EnduroBone bioreactor setup. All files are available as Mendeley repository [31].

**Design file name**	**File type**	**Open-source license**	**Location of the file (DOI)**
Bioreactor-assembly	CAD	CC BY 4.0	https://doi.org/10.17632/w496dkygx8.2
Bioreactor-base	CAD and STL	CC BY 4.0	https://doi.org/10.17632/w496dkygx8.1
Bioreactor-top frame	CAD and STL	CC BY 4.0	https://dx.https://doi.org/10.17632/w496dkygx8.1
Bioreactor-bottom frame	CAD and STL	CC BY 4.0	https://doi.org/10.17632/w496dkygx8.1
Bioreactor-tissue well	CAD and STL	CC BY 4.0	https://doi.org/10.17632/w496dkygx8.1
Bioreactor-motor support	CAD and STL	CC BY 4.0	https://doi.org/10.17632/w496dkygx8.1
Bioreactor-shaft support	CAD and STL	CC BY 4.0	https://doi.org/10.17632/w496dkygx8.1
Bioreactor-cam	CAD and STL	CC BY 4.0	https://doi.org/10.17632/w496dkygx8.1
Bioreactor-follower	CAD and STL	CC BY 4.0	https://doi.org/10.17632/w496dkygx8.1

**Table 2:** Materials used to assemble the EnduroBone bioreactor setup and their cost. Hyperlinks are provided in the table along with component name/no as relevant. The total cost of this setup comes to 3,138.35 USD (*pump alternatives to reduce the cost include open- or four copies of commercial single-channel models, multiple versions Fisherbrand Variable Flow available on Amazon).

**Designator**	**Component/Name**	**No**	**Cost per unit [USD)**	**Total cost [USD]**	**Source of materials**	**Material Type**
Bioreactor assembly, 3D-printing filament	Hatchbox 1.75 mm PLA	1	24.99	24.99	Amazon link	Polylactic acid
Bioreactor assembly, XTC-3D coating	XTC-3D coating	1	30.91	30.91	Amazon link	Epoxy resin
Bioreactor assembly, Slygard-184	24236–10 (Electron microscopy store)	1	199.00	199.00	Amazon link	Silicone
Cam-shaft assembly, stainless steel rod	304 Stainless Steel M6-1.0	1	7.99	7.99	Amazon link	Stainless steel
Cam-shaft assembly, aluminum shaft coupling	BQLZR Blue 6 mm to 6 mm Aluminum Shaft Coupling	1	7.33	7.33	Amazon link	Aluminum
Cam-shaft assembly, lock nuts	M6 x 1 mm Thread 304 Stainless Steel Serrated Flange Metric Hex Lock Nuts	1	5.98	5.98	Amazon link	Stainless steel
Cam-shaft assembly, 12 V 20 RPM DC motor	Greartisan DC 12 V 20RPM Gear Motor	1	14.99	14.99	Amazon link	Other
Perfusion flow assembly (peristaltic pump*)	50–195-3812	1	2,611.20	2,611.20	Fisher Scientific link	ABS engineering plastics
Perfusion flow assembly, platinum-cured silicone tubing	50–195-3823	1	133.73	133.73	Fisher Scientific link	Platinum-Cured Silicone
Perfusion flow assembly, hose barb reducer (1/4″ID x 1/8″ID)	NC0314849	1	39.15	39.15	Fisher Scientific link	Polypropylene
PN2222 Transistor	Adafruit NPN Bipolar Transistors	1	5.88	5.88	Amazon link	Other
270 Ohm Resistor	Chanzon 60 Values 1/4W (0.25 W) Metal Film Fixed Resistor Kit	1	7.99	7.99	Amazon link	Other
1 N4001 Diode	SEMICONDUCTOR 1 N4001 DIODE, STANDARD, 1A, 50 V	1	3.71	3.71	Amazon link	Other
Solderless Breadboard	MCIGICM 400 Points Solderless Breadboard	1	6.69	6.69	Amazon link	Other
Arduino Uno	Arduino Uno REV3 [A000066]	1	24.84	24.84	Amazon link	Other
Dupont Wire	ELEGOO 120pcs Multicolored Dupont Wire	1	6.98	6.98	Amazon link	Other
Orthodontic elastic rubber bands	HRASY 3/16 and 1/4 Inch	1	6.99	6.99	Amazon link	Rubber

## 5 Bill of materials summary

Table 2.

## 6 Build instructions

### 6.1 3D printing and coating of parts

1. Download all CAD files of Table 2.
2. Print all 3D printed components using the design files. There are eight different 3D prints, some of which include multiple pieces. A CAD file for the entire assembly shows all components will come together.
3. Print all parts. We used Ender 5 Plus 3D Printer with a layer height of 0.2 mm, an infill density of 50 %, a print temperature of 200 °C, a bed temperature of 60 °C, and a print speed of 60 mm/sec.
4. Once parts are printed, coat all printed components with XTC-3D sealant to ensure a secure nutrient media seal and prevent interaction between the nutrient media and PLA. Follow the manufacturer’s instructions for mixing and application.
  a. Mix Part A (resin) and Part B (hardener) in a 2:1 ratio.
  b. Apply the mixture as a thin coat to all printed parts, ensuring even coverage for effective sealing.
  c. Leave the parts to cure for 24 h. We placed it inside a biosafety cabinet for curing.
5. All surfaces of the 3D-printed bioreactor were cleaned and degreased using 70 % isopropyl alcohol to remove all surface contaminants. The parts were left to air dry inside a biosafety cabinet before the Slygard-184 application.
6. An additional coating is done to the inside of each tissue well using PDMS Sylgard-184 coating to enhance biocompatibility since they are in direct contact with the tissue and the media, mixed and applied according to manufacturer instructions. The selection of the Sylgard-184 coating for tissue wells was to improve the tissue well biocompatibility based on similar coating applications in other cell culture protocols [25], [34].
  a. Combine the base and curing reagent in a 50-mL test tube at 10:1, stirring for 10 min.
  b. Allow the mixture to set for 30 min before pouring it into the tissue wells resting on a flat surface.
  c. Once the sealant is poured, invert the tissue wells and leave them in that position for curing. This step will allow the sealant to spread evenly along the entire inner surface of the well without pooling or settling at the bottom.
  d. Cure the PDMS-coated tissue wells for three days at room temperature.
7. Clean and degrease all surfaces of the 3D-printed bioreactor using 70 % isopropyl alcohol to remove any surface contaminants and ensure a clean environment for subsequent steps. Allow the parts to air dry inside a biosafety cabinet before proceeding.
8. Rinse the coated tissue wells six times with PBS (Thermo Fisher Scientific). This process is used as an additional safety measure for maintaining a clean tissue environment, following a similar protocol used in the literature ^16^.

Note: Opening the CAD assembly file is not needed in this process. However, if the assembly file is opened, the local directory path of the CAD files needs to be reassigned to SolidWorks to recreate the assembly successfully.

### 6.2 Initial assembly

1. Fig. 5a shows the base platform for a close-up view of the inflow/outflow channels of tissue wells in a linear configuration along the two walls of the bottom frame.

**Fig. 5:**
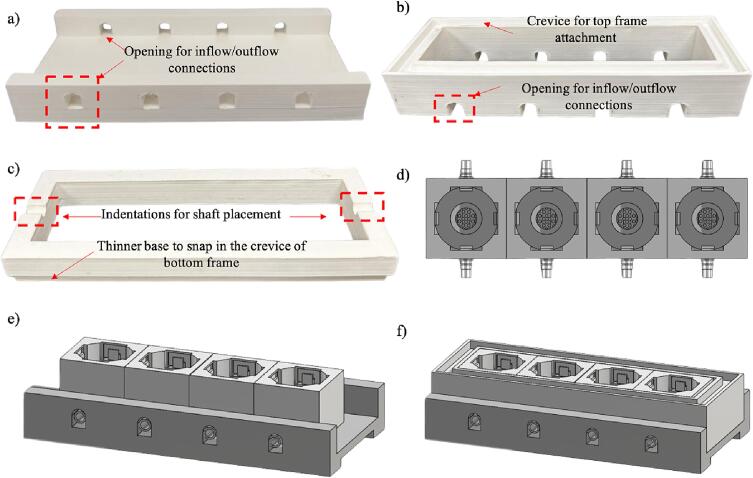
Detailed view of the initial assembly steps: (a) base platform showing channels that are aligning with openings in the tissue well; (b) bottom frame displaying crevice for top frame attachment and opening for inflow/outflow channel of tissue wells, with the crevice allowing the top frame to snap in place and holding a transparent layer of parafilm film to seal in nutrient media during bioreactor operation; (c) the top frame showing two opposing indentations designed for shaft placement and a thinner base for snap fitting in the crevice of the bottom frame, (d) plan view of tissue wells, (e) isometric view of tissue wells, and (f) final aligning and securing tissue wells with the bottom frame. The wells are arranged linearly atop the base platform with the inflow-outflow channels and opening on the base platform aligned for media flow.
1. Fig. 5b shows the bottom frame with a crevice around the top and the extrusion openings at the bottom. The crevice allows the top frame to snap on and holds a transparent layer of parafilm between the two frames to seal in nutrient media during bioreactor operation. The opening at the bottom is for fitting on top of the inflow-outflow channel of the tissue well.
2. Fig. 5c shows the top frame with two indentations on opposite ends for the shaft to rest.
3. Arrange the four identical tissue wells in a linear order on top of the base platform. Align the inflow-outflow channels with the opening on the base platform (Fig. 5d and 5e).
4. Next, place the top frame over the tissue wells to secure the wells in place by aligning the extrusions on the base platform with the top frame. Fig. 5f shows the assembled component at the end of this step.
5. Place rubber bands through two opposite sides of each of the follower holes of the cam-follower assembly to attach them to the hooks on each side of the inner tissue well. This allows the followers to be secured above the tissue sample. Fig. 6a shows the whole four-tissue assembly with each of the followers attached. We used a dental rubber band for this step to keep all parts biocompatible since they are autoclavable.

**Fig. 6:**
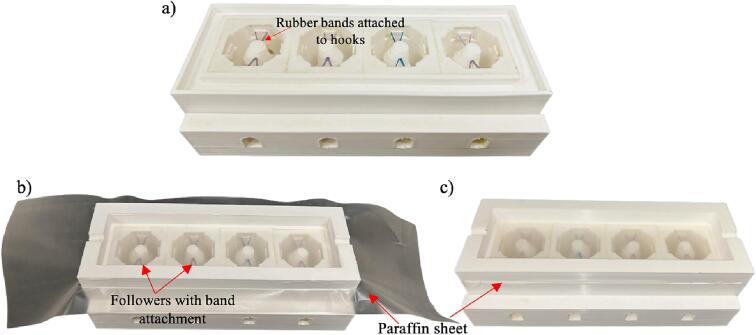
View of the assembly of followers of the cam-follower assembly within the tissue well and placement of paraffin sheet: (a) the four-tissue well assembly with attached followers using dental rubber bands, (b) parafilm enclosure on the top framework before adjustment, and (c) after removal of outer layers using a surgical blade. Please note that in the images shown above, the follower was stabilized on the two sides. Additional stabilization can be added with the bands attached on all four sides.
6. The top frame is surrounded by a crevice to cover the tissue well with a sterile plastic layer, parafilm, to keep the nutrient media sterile. Place the plastic layer over the bottom frame and cover it using the top cover part (Fig. 6b). The film is transparent, but the photo was taken using the black backdrop below the film to demonstrate the placement of the film. Put the top frame and then use a surgical blade to remove the parafilm excess (Fig. 6b). The bottom and top frames of the device are then securely fastened with parafilm film in between them (Fig. 6c).

### 6.3 Motor assembly

**Fig. 7:**
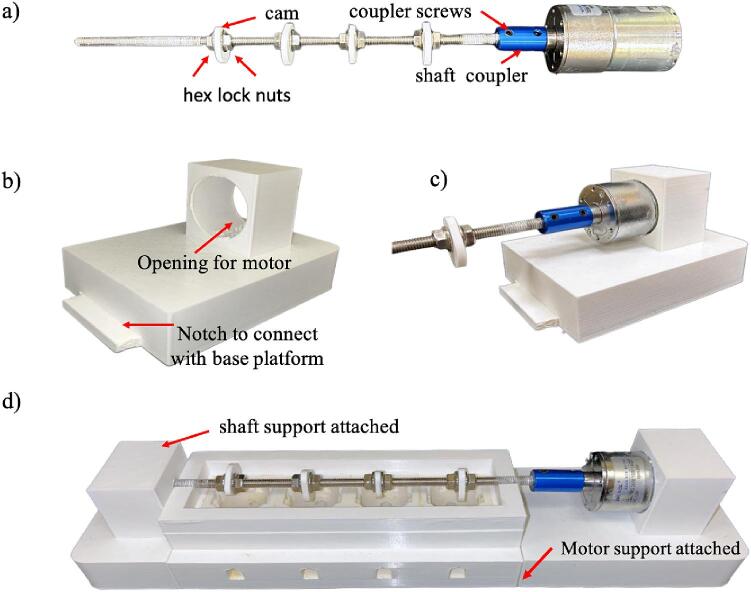
The assembly process for the motor system assembly to the bioreactor: (a) cam and coupler placement on the stainless-steel rod. The coupler is for attaching the DC motor to the rod with screws and hex head spanner, and the cam is attached using M6 x 1 mm stainless steel hex lock nuts; (b) 3D printed motor support component; (c) depiction of proper motor placement connected to cam-shaft assembly into motor support extrusion, and (d) attaching motor and shaft support piece to the bioreactor base platform. The shaft support is needed for cam-shaft assembly stability.
2. Place four cams evenly spaced on the stainless-steel rod and secure them using M6 x 1 mm stainless steel hex lock nuts, one on each side of each cam as marked for one of the cams (Fig. 7a).
  e. The cam-spacing is determined by the 3.8 cm center-to-center spacing of tissue wells.
  f. The cam angles will need manual minor adjustment and visual inspection to ensure the cams are centered directly and perpendicular over the middle of their respective tissue well.
3. Place the motor connected to the cam-follower assembly into the opening of the motor support frame by press-fit. Fig. 7c shows the motor support frame without the motor placed, and Fig. 7d shows the frame after placing the motor.
4. Connect the motor support piece to the bioreactor base platform. Ensure the cams are evenly spaced over the four tissue wells (Fig. 7d).
5. Finally, attach the shaft support to the bioreactor base platform to ensure the stability of the cam-shaft assembly (Fig. 7d).

*Note*: The presence of a white coating on the stainless-steel rod is due to the wear of the PLA material when rubbing against steel, which may require changing the base due to excessive wear. Alternative modifications to increase longevity can include other material combinations, such as PLA/PLA contact by replacing the steel with PLA sleeves or the use of a suitable lubricant. We also recommend using PSU for PLA replacement for the design (See Section 2.2).

1. Attach the DC motor to the 250 mm stainless steel rod using the 6 mm aluminum shaft coupler motor connector. Use four screws per coupler, two on each side, and screw them on using a hex head spanner (Fig. 7a).

## 7 Operation instructions

### 7.1 Powering the motor

1. To power the motor for running the cam-follower assembly, the 12 V DC motor interfaces with a circuit board and an Arduino (Arduino UNO, Arduino.cc), as depicted in Fig. 8, and connects it to the DC motor.

**Fig. 8:**
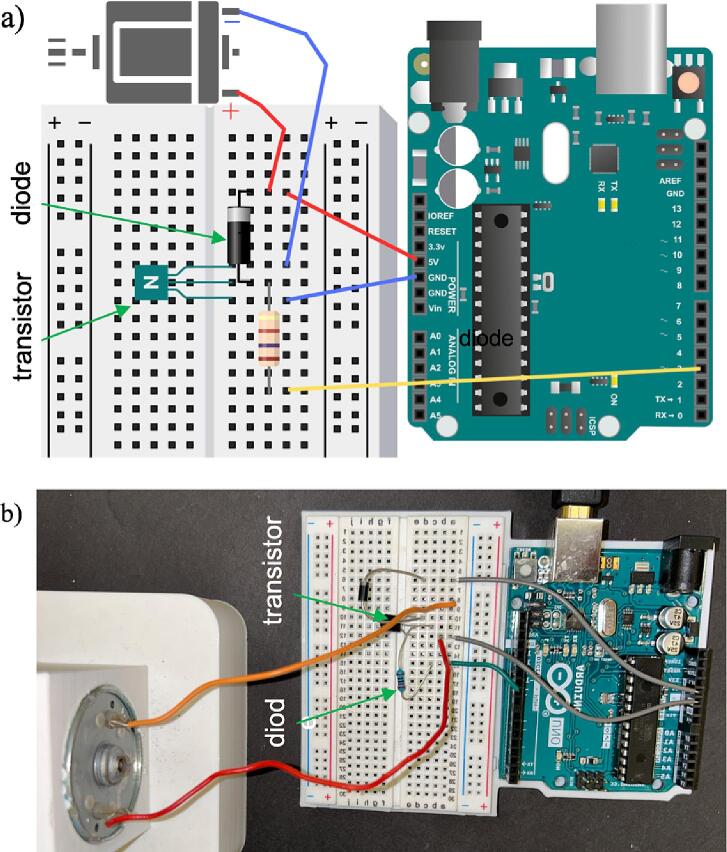
Circuit for powering the 12 V DC Motor with Arduino board showing (a) schematic diagram and (b) as-assembled. Note the orientation of the transistor and diode as indicated in the diagram. The flat side of the transistor is aligned with the Arduino board, and the striped end of the diode is aligned with the designated + 5 V power line.
a. The proper positioning of the transistor involves aligning its flat side with the Arduino board, as shown in Fig. 8. This alignment is essential to ensure adequate connectivity and functionality.
b. To correctly orient the diode, align its striped end with the designated + 5 V power line.
c. The transistor functions as a switch, controlling the motor power.
2. Arduino Configuration for Motor Control

The accompanying Arduino code provided in the Operation Instructions uses pin 9, designated as ’motorPin’ to control the activation and deactivation of the transistor, managing the motor’s functionality. The code and instructions for setting up are below.

a. Go to the Arduino Web Editor website (https://create.arduino.cc/editor) and log in to an Arduino account.
b. 7
c. Click on “Boards” in the top left corner of the Arduino Web Editor. Then click “Add Arduino” and select the specific Arduino board model from the list.
d. Create a new sketch by clicking on “File” > “New” and paste the code provided below. You may need to experimentally adjust the motor speed to achieve 13.2 RPM (revolutions per minute), as the relationship between the RPM and the pulse width modulation (PWM) signal on Arduino is not linear and can depend on the motor type and the power supply voltage.

**Table:** 

*int motorPin = 3;*
*int speed = 172; // Set the speed to 172 (adjust as needed)*
*void setup() {*
*pinMode(motorPin, OUTPUT);*
*analogWrite(motorPin, speed); // Set the initial motor speed*
*}*
*void loop() {*
*// This loop is intentionally left empty*
*}*

1. Click on the right arrow icon (or use the keyboard shortcut Ctrl + U) to verify and upload the sketch to the connected Arduino board. The upload status will be displayed in the lower section of the web editor. The LEDs on the Arduino board may also indicate activity during the upload process.
2. Once the upload is complete, the code will start running on the Arduino board, and the board will power the DC motor system.

*Note*: The above Arduino-based system for motor control is the approach used here. Several other alternatives can also be used for motor control. One alternative will be to achieve a stable system with the breadboard replaced with a PCB. Another more cost-effective option could be directly controlling the motor speed using a PWM (Pulse Width Modulation), which can directly control the electric power to the motor for control.

### 7.2 Force and strain calibration

While the bioreactor can be used without the calibration step, it is important to quantify the load induced on the bone tissue for many applications. The loading profile was calibrated using an off-the-shelf compression load cell (FC22 series, TE Connectivity, USA) with the CellScale Univert Mechanical Test System (CellScale, Canada). Any other tensile system can be used in its place for strain calibration.

1. The load cell was securely positioned on a fixed, perfectly horizontal machine base, and a flat loading platen affixed to a 5 kN load cell (Fig. 9a and 9b),

**Fig. 9:**
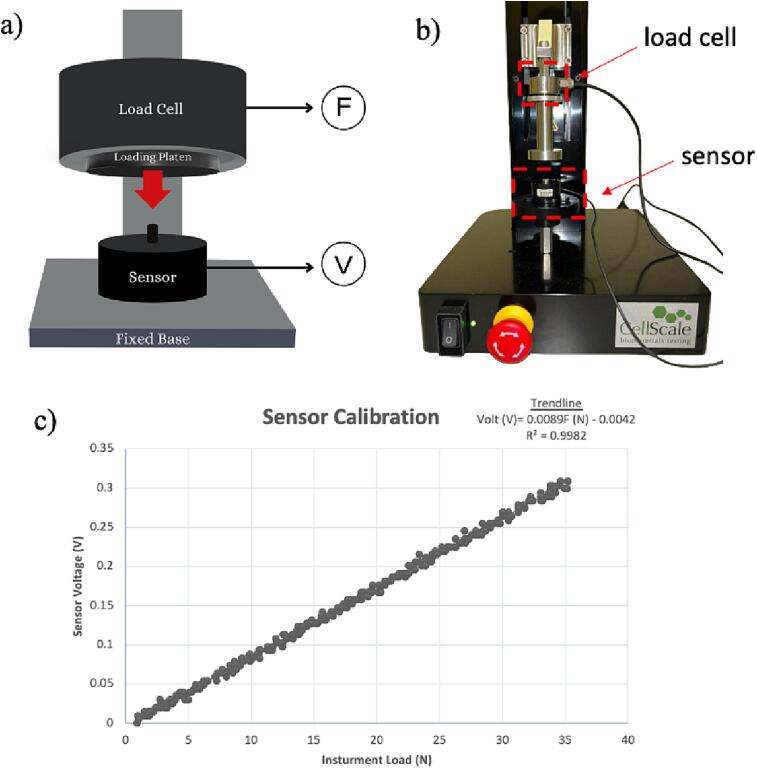
Calibration of the force sensor, showing (a) schematic of the setup, (b) experimental setup, and (c) calibration curve generated depicting the correlation between voltage (V) and force (N) for the load sensor. Force readings were collected by the load cell of the tensile tester and voltage readings were simultaneously collected using Arduino.
2. The loading platen was pressed at a constant speed of 0.1 in./minute. The load–displacement data was recorded in real time using the machine at a frequency of 10 Hz.
3. Additionally, sensor voltage value changes were captured using an Arduino microcontroller (Arduino UNO, Arduino.cc) and a serial monitor.
4. The data collected from both setups was synchronized using time stamps to create a voltage-force calibration curve (Fig. 9c). This curve combined load cell voltage readings with force measurements to create a calibrated relationship between sensor reading V (in volts) and load F (in Newton), as represented by the equation below.
5. The calibrated load sensor was then used to derive the loading profile (force vs. time) applied by the cam-follower assembly. The equation below gives the final calibrated relationship between sensor reading (V) and instrument load (N).

V=0.0089F-0.0042

6. The calibrated load sensor was then used to acquire the force vs. time loading profile applied by the cam-follower setup on the sensor loaded under the tissue well at the same height as the bone tissue. The generated loading profile is depicted in Fig. 10

**Fig. 10:**
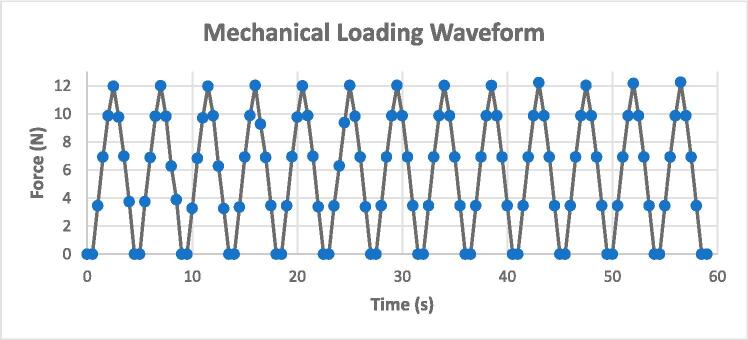
Mechanical loading waveform depicted as Force (N) vs. Time (s) for mechanical loading of the samples.
7. showing a triangular waveform with a peak load of 12 N at a frequency of 0.22 Hz.

It is important to note that from the above force calibration, strain induced on the sample can be estimated assuming a linear relation between stress and strain or Hooke’s law. Literature states that the macro-scale compression stiffness of cancellous bone varies from 20 to 500 MPa [35], [36], [37]. Hence, with 12 N force on a 10 mm core (i.e., area 78.5 mm^2^), the maximum compressive strain is calculated as 7640 με. A similar calculation can be done to estimate the strain environment for the bone tissue being tested by using its value of elastic stiffness. Another alternative is calibrating the distance of cam motion with a material of comparable stiffness to the bone, though the conversion of the cam distance traveled to strain induced on the sample will not be straightforward due to the local deformation of the sample. Finally, by modifying the cam parameters (radius and offset), other targeted values of force/deformation/strain profile can also be achieved based on application needs.

### 7.3 Perfusion flow setup

The setup for perfusion flow followed instructions of the commercial pump, with key steps listed below.

1. Sterilize the platinum-cured silicone tubing and polypropylene hose barb reducers using an autoclave. Use the unwrapped autoclave cycle and the temperature of 121 °C (1.2 bar) for 22 min.
2. Position the tubing inside the tubing retainers. Ensure that the yellow indicators are aligned with the edges of the tubing retainers to secure and maintain the tubing in position while allowing for controlled and consistent flow through the pump system (Fig. 11a).

**Fig. 11:**
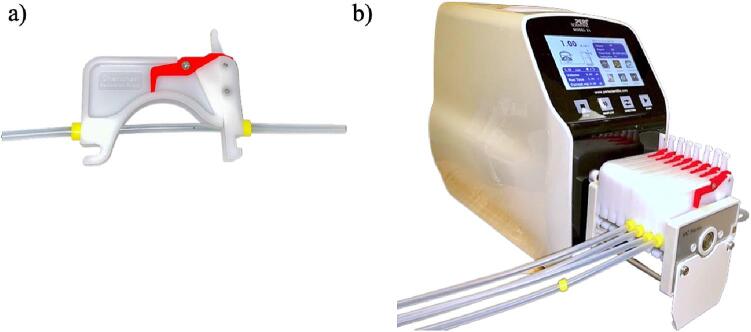
(a) Proper tubing placement and (b) installment of tubing retainers.
3. Attach the platinum-cured silicone tubing to the MC-8 8-channel pump head. Ensure the clamps securely hold the tubing in place without excessively compressing or damaging it (Fig. 11b).
4. Verify the calibration of the perfusion pump to ensure it can deliver the intended 1 mL/min volume.
5. Adjust the perfusion pump settings to a 1 mL/min flow rate.
6. Connect the polypropylene hose barb reducers (1/4″ ID to 1/8″ ID) to the inlet and outlet channels of each of the four tissue wells.
7. Connect the platinum-cured silicone tubing to the polypropylene hose barb reducers (Fig. 12). Since the tubing is connected to the well after it is placed on the platform, the base platform and the bottom frame presence prevent its view in the assembled form. Hence, the figure shows the well independently without the assembly to visualize the proper tubing connection.

**Fig. 12:**
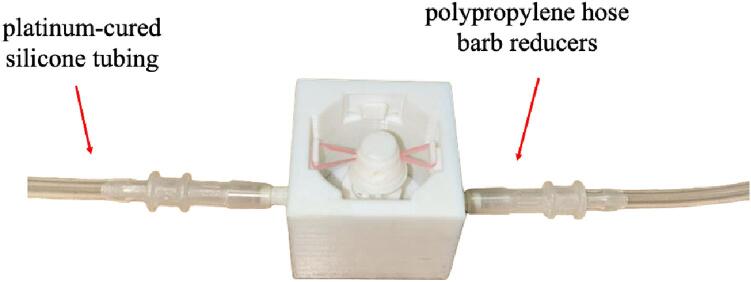
Connection process of polypropylene hose barb reducers to inlet and outlet channels, followed by attachment of platinum-cured silicone tubing in the four tissue wells.
8. Once the tissue wells are connected to the tubing and the samples are positioned in each tissue well, introduce the pre-prepared nutrient media into each well.
9. Start the peristaltic pump to initiate the perfusion flow.

### 7.4 Mechanical loading setup

Before proceeding with the mechanical loading process, ensure that you have successfully assembled the components according to the assembly instructions provided. The mechanical loading process involves cyclic compression of bone samples using a cam-follower assembly. This assembly exerts periodic pressure on the samples, inducing mechanical loading to simulate physiological conditions. A 12 V DC motor powers the process interfaced with a circuit board and an Arduino board.

1. Ensure the assembled components are securely in place, including the cam-follower assembly and the motor support.
2. Confirm that the cams are evenly spaced over the four tissue wells on the bioreactor base platform.
3. Power on the system by providing the necessary power supply to the 12 V DC motor.
4. Launch the Arduino code on the Arduino board to control the motor’s activation and deactivation.
5. Once the system is powered on, allow the motor to spin for one hour.
6. Observe the cyclic mechanical loading process as the motor engages with the cam-follower assembly. The rotation of the cam generates periodic pressure on the bone samples.
7. After one hour of mechanical loading, safely power down the 12 V DC motor.

*Note*: Ensure proper connectivity of the 12 V DC motor with the circuit board and the Arduino board, as shown in Fig. 9. Position the transistor and diode according to the orientation specified in the figure for proper functionality. Also note that we secured the follower of the cam-follower assembly with rubber sides attached on two opposite sides, though the design has hooks on all four sides. So, additional stabilization for uniform loading can be achieved with the bands attached on all four sides.

## 8 Validation and characterization

A study was conducted to investigate the changes in cell viability over 28 days, following an earlier similar work from the lab on long-term tissue survivability [23]. Samples of tissue were taken from the culture wells at specific time intervals (Day 0, 7, 14, 21, and 28).

### 8.1 Sample preparation

The specimens used in this study were sourced from a prior IACUC-approved study led by the University of Miami Veterinary Department. They were not specifically obtained for this research but were graciously donated as a surplus from an existing study.

Fresh femoral sections were obtained from swine specimens within one hour of their passing and were transported in an ice box to prevent contamination or degradation of cells. The samples were then cleansed by rinsing them with deionized water to remove any loose debris and eliminate soft tissues. A 4-inch segment of the femoral head was extracted using a bandsaw, and cylindrical cores with a diameter of 10 mm were extracted using a diamond-tipped hollow fluted drill. These cores were then sectioned to a thickness of 10 mm using a high-speed saw while maintaining a continuous irrigation process with a 0.9 % NaCl solution at 4 °C to prevent cell death. Multiple such cores were extracted to create replicas for loading and time-point extraction for testing. These extracted cylindrical cores were placed inside the tissue well with raised edges to hold the bone samples in place while loading.

### 8.2 Nutrient media and waste removal

Freshly harvested swine bone cores were immersed in the tissue well and stored in an incubator. To do so, the bone tissue samples were first carefully positioned in their designated wells within a sterile biosafety cabinet to ensure aseptic conditions. Once confirmed to be securely in place, the samples were enclosed by the parafilm stretched across the base of the bioreactor and secured by the top frame. Following the parafilm enclosure, the fully assembled bioreactor containing the bone samples can be transported to the cell culture incubator, and its inlet and outlet ports are connected via sterilized tubing to the peristaltic pump located outside the incubator. A flow rate of 1 ml/min of the pre-prepared nutrient media was initiated. The temperature was set to 37 °C and the CO2 density to 5 %, while the media was kept slightly basic to mimic the native environment that promotes bone growth [38].

The primary nutrient media was serum-free Dulbecco’s Modified Eagle Media (DMEM) (Life Technologies Corporation), with the addition of Penicillin and Streptomycin (Life Technologies Corporation) as antibiotics to prevent bacterial contamination. [39] GlutaMAX (Life Technologies Corporation) was also used to support higher growth yields, more efficient metabolism, increased media stability, and reduced toxic ammonia build-up. [40] HEPES (Life Technologies Corporation) was used as a buffer to maintain the media’s pH due to its ability to maintain physiological pH despite changes in CO2 concentration produced by cellular respiration [41]. Fetal Bovine Serum (Thermo Fisher Scientific) was included as a rich source of amino acids, proteins, vitamins, carbohydrates, growth factors, lipids, and hormones to support bone growth further. L-ascorbic acid (Sigma-Aldrich) acted as an antioxidant, improving cell survivability, while Zinc chloride (Thermo Fisher Scientific) contributed to increased bone cell proliferation and collagen synthesis. [42] To optimize the culture conditions and ensure the success of the experimental model, the B-27 supplement (Life Technologies Corporation) was included in the nutrient media [43]. To prepare the nutrient media, 25 mL of DMEM was combined with 250 µL of Penicillin-Streptomycin solution, 100 µL of GlutaMAX, and 250 µL of 1 M HEPES in a sterile container. Then, 2.5 mL of Fetal Bovine Serum, 1.25 mg of L-ascorbic acid, 25 mg of Zinc chloride, and 500 µL of 50X B-27 supplement were added. All the components were gently mixed to ensure even distribution. The freshly harvested swine bone cores were placed into tissue wells and immersed in the nutrient media. The entire setup was stored in the incubator under the previously specified conditions. The perfusion flow setup maintained the closed-loop flow at 1 ml/min, the value based on earlier work at finding the optimum flow rate [23]. The nutrient media was replaced every three days with fresh media using the same quantities and components to maintain optimal conditions for bone growth.

Since the *EnduroBone* bioreactor is a closed-loop system with a peristaltic pump, waste removal occurs through the continuous cycling of nutrient media, with the peristaltic pump ensuring a consistent flow. As mentioned, the media was replaced every three days to maintain optimal nutrient levels and remove accumulated waste. To maintain sterility during the entire process, we used two sets of autoclavable polypropylene tubes for the nutrient media. After 3 days of use, the tubes were replaced with another set filled with the nutrient media as described above. Sterility was maintained throughout, with the tube being autoclaved prior to filling and the transfer occurring within the biosafety cabinet.

### 8.3 Mechanical loading

The cyclic loading was applied once daily for one hour each day over the course of 28 days. Section 4.1 describes powering the motor at 13.2 RPM. The loading was achieved by keeping the Arduino board inside the incubator alongside the bioreactor. We still used the 5 V DC power, but the USB cables were disinfected using 70 % isopropyl alcohol wipes before entering the incubator. The incubator door remained closed during the culture period, except for the times when samples were extracted, and nutrient media was replaced, which helped minimize the risk of airborne contamination from outside electronics. Furthermore, since our bioreactor has a closed-loop nutrient flow and a seal on the top of the tissue well, the whole system does not come in contact with the outside and remains sealed through this process. As an additional precaution, we enclosed the Arduino board with breadboard inside a 3D-printed PLA Arduino holder with a cover, which was sterilized using 70 % isopropyl alcohol before placing it inside the chamber. The housing design was printed using publicly available CAD files [44]. Alternatively, the Arduino board can be kept outside with the use of a longer wire for this process.

### 8.4 Cellular viability assessment: methodology and imaging

The assessment of cell viability is a crucial parameter in evaluating long-term survival in various biological systems. Live/Dead cell staining techniques have been widely used for this purpose [2], [45]. Based on these, in this study, we utilized the Calcein AM/Ethidium homodimer-III (EthD-III) (Viability/Cytotoxicity Assay Kit for Animal Live & Dead Cells, Biotium) dyes to evaluate cell viability in extracted samples. The confocal microscope can be used to image the samples’ exposed internal cross-sections after staining. The staining signals of Calcein AM and EthD-III can be detected using specific excitation and emission filters. This technique can be utilized for various biological systems, providing a reliable and accurate assessment of cell viability.

Once every 7 days, one sample was taken out of the system and examined under a confocal microscope. These samples were not returned back to the system. To prepare the samples for staining, extracted samples underwent triple washes with sterile PBS to remove residual debris and nutrients. The cylindrical samples were then longitudinally sectioned to expose core cells. A staining solution was created by combining 2.5 μL of 4 mM Calcein AM with 10 μL of 2 mM EthD-III in 5 mL of sterile PBS. This resulted in a 2 μM Calcein AM/4 μM EthD-III concentration, which was then applied and incubated in the dark at room temperature for 45 min. The samples were then subjected to another round of washing with sterile PBS.

Calcein AM/Ethidium homodimer-III (EthD-III) was used for live/dead cell staining. Calcein AM dye permeates cell membranes, generating green fluorescence upon enzymatic cleavage in live cells. EthD-III, penetrates dead cells with compromised plasma membranes, specifically staining the nucleus with vivid red fluorescence. A specialized sample holder featuring extrusions to house a 3D bone was utilized to image the samples. Thin glass coverslips and duct tape facilitated adherence to the customized holder. The exposed internal cross-sections of the samples were promptly imaged under a confocal microscope after 45 min of staining in the dark. Calcein AM exhibited an excitation/emission of 494/517 nm, while EthD-III displayed an excitation/emission of 530/620 nm. Excitation and emission filters specific to each stain were employed for their detection, resulting in green and red colors corresponding to live and dead cells, respectively.

**Fig. 13:**
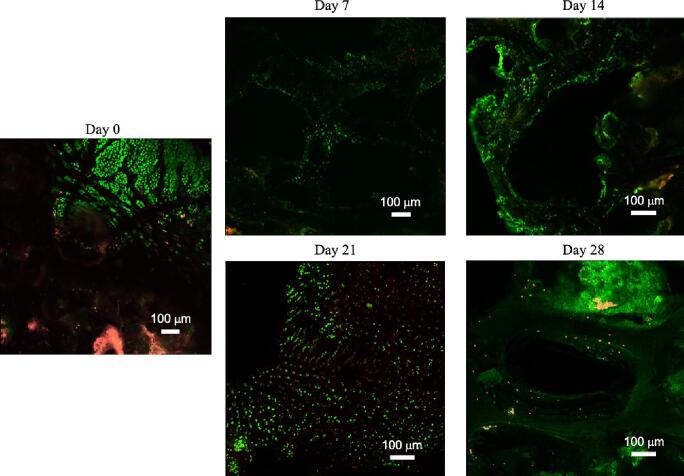
Temporal assessment of cell viability using confocal microscopy: live/dead cell assessment at Day 0 vs post tissue culture in the bioreactor (Days 7, 14, 21, and 28).

The ex-vivo viability of the bone core samples was maintained over the 28-day experiment, as evidenced by the low proportion of dead cells observed in the bioreactor group samples (Fig. 13). These findings were consistent with the results from the Day 0 bone core, which was used to confirm cellular viability. By continuously circulating nutrient media through the bone samples, waste was effectively removed, and dynamic hydrostatic pressure was generated on the tissue. Periodic mechanical loading is known to stimulate the osteocytes to promote bone remodeling activities, which can further contribute to improving cellular viability. These responses on the bone matrix can be quantified through detailed testing, such as nanoindentation for mechanical performance and Raman spectroscopy for matrix composition. The outcome of such detailed tissue analysis was demonstrated in a separate study from our group using an early iteration of the bioreactor that guided the current design ^14^, which can form the basis of future detailed analysis. Overall, the ex-vivo bioreactor system *EnduroBone* maintained the viability of the bone cells throughout the study due to a combination of continuous nutrition delivery and mechanical stress.

## 9 Summary and conclusion

This paper describes the design and validation of a new Bioreactor *EnduroBone*, precisely designed to support 3D bone tissue while ensuring prolonged cell viability over the long term. Unlike conventional tissue culture setups with only perfusion, our bioreactor integrates a motorized cam-follower assembly alongside precise perfusion flow mechanisms, thus providing an integrated arrangement of nutrient delivery within a dynamic loading environment to mimic the physiological environment accurately. Furthermore, through cell viability experimentation, we have demonstrated the resilience of our cultured tissue for up to 28 days, a testament to its device performance. A distinguishable feature of the bioreactor design is its potential for multifaceted utility across diverse domains of bone engineering research beyond its foundational role in long-term tissue preservation. For example, it can serve as an invaluable tool for clarifying the complex interactions and communication among various bone cells and how outside stimuli can affect the dynamics of such interactions. This versatility of the bioreactor could extend beyond bone research to advance cartilage research by examining cartilage degradation over time and observing the transition from cartilage to bone throughout growth by growing new cartilage tissue.

Overall, by offering a controlled environment to explore the influence of external stimuli on bone tissue and related cellular activity, the *EnduroBone* system could enable research in various fields that could benefit from an ex vivo bone culture platform, and it is a beneficial method for better understanding pathophysiological mechanisms underlying bone biology and complex mechanisms governing skeletal development and regeneration. Beyond these immediate applications, it can also support transformative advancements in regenerative medicine and in the development of novel therapeutics and interventions to combat a spectrum of bone-related ailments and injuries.

## CRediT authorship contribution statement

**Paula Gustin:** Writing – original draft, Visualization, Validation, Methodology, Formal analysis. **Anamika Prasad:** Writing – review & editing, Visualization, Supervision, Resources, Project administration, Methodology, Funding acquisition, Conceptualization.

## Declaration of competing interest

The authors declare that they have no known competing financial interests or personal relationships that could have appeared to influence the work reported in this paper.
